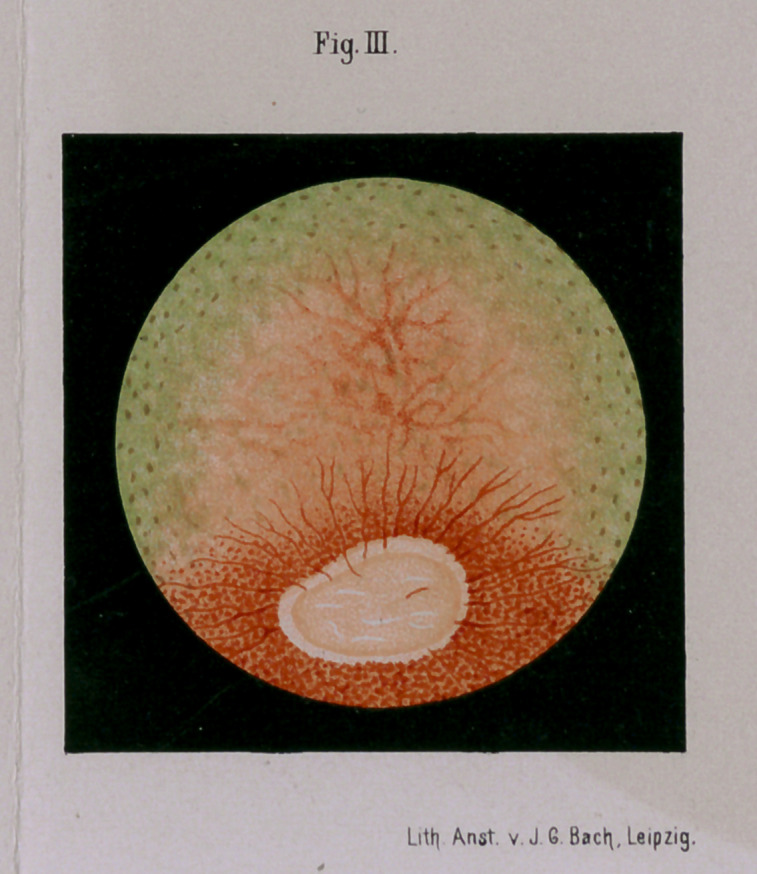# The Ophthalmoscopic Appearance of the Fundus of the Eye of the Horse

**Published:** 1884-01

**Authors:** 

**Affiliations:** Stuttgart


### Art. V.—THE OPTHALMOSCOPIC APPEARANCE OF THE FUNDUS OF THE EYE OF THE HORSE.

BY PROF. DR. BERLIN OF STUTTGART.

THE opthalmoscopic appearance of the interior of the Eye of the domestic animals has been a study of great interest to me, and one which I consider of great practical importance.

Aside from the numerous anatomical variations which appear in the eyes of the different species of animals, the individuals present so many divergences in form and color that it requires no inconsiderable degree of experience to draw a correct line between that which is common or uncommon, and that which is normal or pathological in the pictures presented to our inspection.

A certain degree of attention in describing these conditions would have saved observers from many errors in diagnosis.

It is our intention to enter into detailed descriptions of the opthalmoscopic appearances of the interior chamber of the eye of the different domestic animals, in both normal and pathological conditions, in so far as they have a practical value, beginning with the eye of the horse in a normal condition.

Both patience and perseverance are necessary on the part of the observer to make a careful and correct study of the eye of the horse on account of the uneasiness and resistance which the animal frequently offers. This uneasiness varies greatly in the different animals; some are perfect examples of docility while under examination, and are thus especially adapted for study and class demonstrations; others keep their heads nodding all the time, some again keep the eye in motion by contraction of its muscles, and these movements seeming to be mostly in a horrizontal direction, with an outward and upward inclination.

Some animals have an especial inclination to close the eye on application of the opthalmoscope. If the eye-lashes are too long and interfere with the use of the instrument they may be cut off. Occasionally an animal will be found that most obstinately closes its eye on every attempt at an examination. If in such a case a correct diagnosis is an absolute necessity, the lids must be kept apart by the fingers, or instrument, and the head confined by the application of the "twister," or other mechanical means; sometimes the "nicatans" is brought over the cornea in such a way as to most seriously interfere with the examination; still I can remember but few cases in which I have been unable to make a satisfactory examination during my nine years study of this subject. Any animal that forcibly resists the separation of the lids and the examination is unsuitable for class demonstrations.

The examination can be made either by natural or artificial light; by the first it is necessary that the light come through a small opening; the larger this opening is, the more it disturbs the regularity of the opthalmoscopic picture. Artificial light is, however, preferable for instruction because it can always be had and is more easily regulated. Veterinary schools and hospitals should have a dark chamber or stall with a gas apparatus suitable to the purpose. In practice, the observer will always have to adapt himself to circumstances, nevertheless one can soon become expert in the examination by continued practise. It is however necessary that students should be exercised in the use of daylight so that they become accustomed to the variations in shade which may be observed between natural and artificial light. The descriptions here given have all been made by artificial light.

Is it advisable to use mydriatics to dilate the pupil before examination or not? Experience justifies us in asserting that this procedure *is* advisable; it lessens the disturbing influence of the corneal reflexion, increases the clearness of the fundus of the eye and enables one to recognize pathological attachments of the iris more readily and renders more accessible to our vision the entire interior of the eye, by increasing the field of observation. On the other hand the dilatation of the pupil by atropia does not cause any disturbance worthy of mention. In dogs and cats, some of the solution sometimes gets into the pharynx by means of the lachrymal duct, causing an increased flow of saliva for a few moments, a circumstance I have never yet observed in horses, neither that it exerted any disturbing influences upon their sight. It appears therefore that we need have no fear of injury to the *normal eye* from the use of mydriatics in our examinations, yet I must bear witness to unfortunate results which happened to a colleague from the use of the same. A mare heavy with foal was handed over to his care by the owner, but not for treatment in any way. The Veterinarian accidentally observed that the left pupil was almost closed a small clear spot on the inner portion of the same enabled him to see that the lens was clouded. Having a scientific practical interest in the study of such questions he placed a few drops of atropia solution in the conjunctival sac of the eye in question which caused the synechia (attachments) between the capsule of the lens and iris to rupture, and contraction of the latter so that the condition (cataract) of the lens could be thoroughly seen. The iris did not retract as was expected notwithstanding the use of eserine as a nyrtic. The owner next day, observed the eye, and was very angry at what had been done, considering that a hitherto unknown blemished and unsoundness of his horse had been revealed by the uncalled for action of the practioner; he did not carry the case to court however, yet it should be a warning to practitioners to have the owners consent before making such experiments, except as expert examiners in forensic cases. With practice, however, the use of atropia may be dispensed with, yet it should always be used for instructing students on account of the many advantages which it offers. In order to examine the eye of a horse, the animal must be brought into a dark cabinet, the pupil having been dilated by a few drops of one per cent solution of atropia placed in the conjunctival sac about half an hour previously. The head of the horse must be kept in position by attendants; one of whom should hold the light upon the side we do not wish to examine somewhat higher than the level of the eye and about a foot to the side of it. The observer should place himself forward and a little oneside the eye to be examined, and place the opthalmoscope over his right eye, so that the reflected light shall fall into the eye which he wishes to study. As a rule I use an instrument having a diameter of 43 mm. the central opening being 3.5 mm. and the focal distance about 7 inches. I always use a plane lens, which gives an upright picture, the transposed picture of the convex lens being so small as to be too indistinct for our purposes; by the latter we can scarcely see the smaller vessels. One must approach within about 10 cms. of the eye in using the plane lens.

When we look into the horses eye in the manner above described, it presents to view a reddish color, which Föringer has described as more nearly resembling that of a blood-orange than anything else.

The shades vary considerably and have been described as follows: "The external limits of the disk of the optic nerve are of a very clear whitish yellow color followed inwardly by a smaller zone of yellowish red, then one of whitish yellow with a tinge of red in it, the centre of the disk being more red, its regularity being interrupted by a number of roundish spots of uncertain color." According to my observations the width of the external, or whitish ring in its superior part is about one-twelfth that of the entire optic disk papilla; it is narrower in its interior portion and of a lighter color, due to a greater accumulation of pigment in this section of the fundus of the eye. This pigment is not situated alone in the epithelium of the retina and choroidea, but I have also found it between the nerve-bundles occupying the peripheral portions of the disk. Sometimes we find imperfections in the continuity of this portion of the disk. We will now consider the middle or more transparent ring of the optical disk which follows immediately upon the reddish portion of the external.

When we have before us cases of atrophy of the optic nerve (amaurosis) we can easily distinguish the outlines of this ring which forms a sort of wreath with more or less ray-like processes extending into the external zone. I have once seen this configuration in a very distinct manner in the normal eye; as a rule, however, in the normal eye they are only indistinctly present.

In atrophy of the optic nerve the central portion of its disk presents a whitish field, the continuity of which is frequently broken by spots, giving it a sieve-like appearance. When we look upon the disk of the optic nerve in an atrophied condition, the picture which it presents to our view by its different portions strongly resembles the appearance of a radish when cut through in an oblique direction.

These results of opthalmoscopic study exactly corresponds with the distribution of the connective-tissue framework of the head of the optic nerve, the so called "Lamina cribosa."

The typical atrophy of the nervous opticus in the horse is also characterized by the general atrophy of the vessels of the bulb of the nerve; hence the color of the latter is in general of a grayish white shade, clearer, where the thicker bundles of connective tissue of the lamina cribosa lie, more gray at such places where the atrophied nerve fibres and vessels are.

The opthalmoscopic appearances of the bulb of the nerve are entirely different under normal conditions. Here we see before the lamina cribosa a considerable layer of fibres of the optic nerve which bend round into the retina and are accompanied by a richly developed rètè of the smallest arteries and veins. The smallest vessels of this rètè are not to be individually distinguished by the opthalmoscope, but their richness in blood gives to the optic disk, as a whole, its reddish yellow color, which is only interrupted at such places where the thicker bands of connective tissue and the lamina cribosa reflect through. Those vessels of the optic disk which are to be distinguished by the opthalmoscope have a specific arrangement and course in the horse. Biervliet and Van Rooy's description of them leaves but little to be desired and is as follows: "The vessels, or better their branches, some twenty or thirty in number, all come out at a certain distance from the centre of the papilla. A central vessel has never been observed. We have indeed seen a large vessel comming out very near the centre of the papilla some three or four times, but it bore no resemblance to the central vessel which is to be seen in man. Most of the vessels of the papilla have a serpentine course, many of them showing spiral windings, but as soon as they leave the papilla their course is in general straight. They become dispersed in the different regions of the retina giving off occasional, generally dichotomous, branches which can be followed for some distance by the opthalmoscope. We have never yet been able to positively distinguish the veins from the arteries probably because of the small calibre of the vessels."

I will remark, however, that in every case where I have subjected the eye of the horse to opthalmoscopic study that I have seen several dark red spots in the centre of the disk, which have also been described by Föringer and Eversbusch, who look upon them as having some relation to a central vessel, while Biervliet, V. Rooy and Esberg consider them of a pathological nature. The size, form and number of these spots is variable as well as the intensity of their color. If there is but one present it may be one-tenth of the perpendicular diameter of the papilla in width, and may be either round, oval, or irregular in form. The greater the number of these spots the smaller they are and the further are they situated from the centre of the optic disk. My own opinion is that these reddish spots represents *knees*, or the points of curves, of the centrally situated vessels which reflect through the layers of nerve and connective tissue of the head of the optic nerve. As the branches of the vessels are, however, given off at different depths it is plain that in some cases we see more of these spots than in others, according to the thickness of the layers of tissue covering them; as we have said the more there are of them the further are they situated from the centre of the disk and the more intensively are they colored red: the deeper situated the vessels are in the body of the nerve, the thicker the layers of tissue covering them. All authors are united in considering the papilla of the optic nerve in the horse to be flat or oval in form. Eversbush considers its transverse diameter to be in proportion to its perpendicular, as 3 : 2. Variations in either direction are frequently met with. Although Föringer describes regular round papilla I have never yet seen one, the horizontal (transverse) diameters always appearing to me to predominate. With Boyer, I have always found the inferior contour of the papilla perceptibly flattened. The irregularities of the contours of the optic nerve, which according to Föringer appear at times very marked, are only optic illusions produced by the irregular astigmatismus of the lens as noted by me in 1879 and since then confirmed by Hirschberg.

All authors unite in describing the surface of the papilla as smooth with the exception of Hisrchberg, who assumes the presence of a central excavation. I have never been able to see any such condition in my examinations. Anatomically speaking, the centre of the papilla is frequently seen to be situated slightly deeper than the circumference of the papilla, but on *excavation*, in an anatomical sense it is not. The vessels seldom appear upon the surface in the centre of the papilla.

Accumulations of pigment in the form of striæ, and rings of more or less completeness are frequently observed in the vicinity of the optic nerve. I have in my possession two copies of the opthalmoscopic picture of the fundus of an eye in which these circumscribed pigmentations are very marked, while the balance of the fundus is almost free from pigment. If this pigment is seated in the chorioidea or the inner layers of the sclera, I cannot say.

Eversbusch has observed the absence of pigment on the immediate periphery of the papilla.

I have seen in two cases in a normal fundus and unaccompanied by disturbances of the sight, a peculiar bluish white appendage attached to the papilla which was about one-tenth to one-eight the size of the latter; at one time this appendage was situated to one side of the papilla, at the other on both sides. It appeared to me to be made up of double contoured nerve fibres a phenomena frequently seen in negroes and sometimes in white people; Kolliker and H. Mueller have found them in anatomical studies of the eye in the ox and dog.

Biervelt and V. Rooy give the following description of the opthalmoscopic appearance of the other portions of the fundus of the eye in the horse.

"The tapetum extends in an anterior superior direction from the optic papilla and is to be distinguished by its variations in color more to be compared to rain-bow hues than anything else. The tapetum is traversed by vessels taking their origin at the optic disk. We have been unable to distinguish the stellate vessels of the "chorio-capillaris" which have been described by several authors, more recently by Eschricht and Brücke. The anterior-superior portion of the tapetum presents an appearance reminding one of the meshes of a net lying upon, and covering, its deeper seated tissues; in the vicinity of the papilla we may see a number of clear, circumscribed points. As we approach the optic disk (papilla) we observe that the color of the tapetum changes from greenish to a bright refracting orange-red, which especially predominates in the immediate vicinity of the nerve and changes to a dark brown or almost black color in the inferior portion of the retina. These variations in color have an intimate connection with the color of the hair of the animal in question. The fundus of the eye is clearer and of a more brilliant red in horses in which white hair predominates, the whiter, the clearer the coloring of the tapetum; the color of the inferior portion of the retina is marked in dark bay horses having black or dark brown hair."

The tapetum occupies somewhat less than two-thirds of the fundus.

Eversbusch describes the "tapetum fibrosum" of the horse as follows: "That part of the choroidea occupied by the tapetum resembles a triangle more or less, the base forming a direct line, while the sides are convex and approch round the apex of the triangle with more or less of a curve. The base of the tapetum comes in a direct line with the superior edge of the papilla: occasionally the tapetum begins from 0.25: 0.5 mm. above the superior edge of the papilla. In very rare instances we find the base of the tapetum embracing the superior half of the optic disk. The extreme vertical dimensions in its median line are from 32.5: 37.5 mms; the greatest diameter is at its base where it is only 2.5: 2.7 mms. distant on either side from the "ora serata;" superiorly it is about double that distance from the latter."

When we compare the opthalmoscopic picture of the visible part of the tapetum, with the above anatomical description of its dimensions, we find the former to be considerably smaller. It is therefore necessary to assume that certain anatomic portions of the tapetum are beyond the limits of opthalmoscopic observation, the reasons for which we shall consider later.

The normal ground-color of the tapetum in the eye of the horse is given by opthalmoscopic observation. When the observations are made with daylight, the green has a somewhat bluish shade. This green or blue-green color of the tapetum is not always evenly distributed over this portion of the choroidea, but is darker or lighter in shade in different parts. The point like spots which we have described vary in size, and are sometimes confluent, especially in the superior and lateral limits of the tapetum. These spots must be looked upon as groups of pigmented cells belonging to the retinal epithelium; the latter contains in general but little pigment in such portions of the tapetum as are visible with the opthalmoscope. The description of the tapetum is about completed by adding to the above the fact that the true limits of the tapetum are almost free from vessels, a few only being visible in its inferior portion; the same are to be easily distinguished from the much darker appearing retinal vessels, while the more profoundly situated chorioideal vessels are to be seen in the superior and lateral portions and distinguished by their red color.

I say "about completed," for it is impossible to accurately describe all the beautiful shades in color presented by that wonderful organ—the tapetum; further, we cannot yet say that the limits of the anatomical study of this portion of the eye have as yet been attained. In order to arrive at limits of what we should consider a normal tapetum it is important that we should study some of its congenital anomalies, the same being all the more interesting as they give to us the physiologic-optical explanation for the opthalmoscopic ground-color of the tapetum.

1. An anomaly, which is, however, but slightly abnormal, consists in the fact that the surface does not present its usual uniform greenish appearance, interrupted only by the previously described dotting, but that it is broken by a net work of coarse striae crossing one another in every possible direction; between these meshes may be seen red spots of varying dimensions which are the vessels of the chorioidea appearing through the tapetum; sometimes we can see the stem of a vessel of considerable size.

2. Another anomaly is that we sometimes see an extensive interruption of variable size in the continuity of the tapetum in one place or another, frequently above the optic nerve; at such a spot the fundus appears red and a number of vessels of variable dimensions may be seen. Fig. 2.

Such interruptions in the continuity of the tapetum are frequently met with in horses that have otherwise nothing abnormal in the color of their hair or eyes. They appear to especially prevail in piebald horses or those having the so-called watch eye; in such the opthalmoscope sometimes fails in revealing any tapetum.

In the last six watch eyes which I have examined, I have found the following abnormalities:

1. Black horse: two watch eyes: in the left eye the want of pigment in the iris was more marked than in the right. The fundus of the left eye presented a uniform red color with choroideal vessels shining through: all indications of a green color failed. In the right eye on the median side a small zone of green tapetum.

2. Red-roan: partial watch eye on each side: tapetum failed in the left; in right a slight green shade in the lateral superior portion of fundus.

3. Piebald white: to the left a complete watch eye, right partially so. Tapetum present in the middle and to the outside of fundus of both eyes of golden yellow color; it was wanting in the superior part where a red reflexion was visible.

This case forms the transition to the third anomaly, which is characterized by a white, or yellowish white color, in place of the greenish usually occupying that part of the fundus to which is given the name tapetum. This white ground presents the normal net work, or points, of the tapetum, but the same are red. This result has *only* come to my observation in watch eye; once in a clear white horse. It can extend over the entire region occupied by the tapetum, or be only partial. As a rule it is united with the anomaly described in "2" so that at certain places, especially peripherally, we can distinguish the red color of the choroidea and numerous vessels.

We have mentioned a number of forms in which the fundus of the eye appears red at the places where continuity of the tapetum is broken in the opthalmoscopic picture, and where the choroideal vessels may be seen reflecting through the non-pigmented tissues: in other cases we may see a whitish or yellowish layer which is only interrupted by numerous reddish spots or lines. The white place represent the "tapetum fibrosum" penetrated by small vessels coming in a direct course through it; behind these spots the choroidea is also wanting entirely in pigment; the layers of the "tapetum fibrosum" are however, so thick that they do not permit the parts posterior to it to reflect through; but on account of the great refraction of its own tissues becomes itself visible.

When the tissues of the tapetum are very thin, we may see the blood-rich choroidea with its large vessels reflecting through as we have already mentioned. It is the same anatomical cause, the continual decrease in the thickness of the "tapetum fibrosum" towards its peripheries, which renders indistinct the lateral and superior limits of the tapetum, the reddish shade of the choroidea reflecting through, while indeed thin layers of the "tapetum fibrosum" are still beyond the limits marked by the optholmoscopic picture.

We have previously described the situation and form of the inferior portion of the tapetum. Its genesis is due to the fact that the epithelial cells of the retina which are but very slightly pigmented and which corresponds to the "tapetum lucidum," become suddenly filled with pigment, which prevents the light from gaining access to the deeper layers of the chorioidea. If we scrape off the epithelium from that part of the fundus which lies directly under the limits of what we may call the optholmoscopic tapetum, we shall see that the "tapetum fibrosum" lies some mms. deeper than the opthalmoscopic tapetum, and in layers of considerable thickness.

The limits of the inferior portion of the tapetum are sometimes well defined, at others indistinct. Sometimes we may see sharply marked spots in that part of the choroidea not occupied by the tapetum; between the latter and the papilla and somewhat to the side of the same, and more inferiorly situated than the lower edge of the papilla, and in eyes having nothing abnormal in their sight, or any pathological, disturbances. They are often present twenty or thirty in number, and vary much in size and form. They are characterized by having the same green color as the adjoining tapetum, and are in general circumscribed by dark peripheries. These spots have been mistaken for evidences of "chorioiditis disseminata;" a mistake which can be easily avoided when one takes into consideration the green color, situation, number, and especially the fact that all inflammatory phenomena are wanting at the time. I hold the same to be isolated portions of the tapetum. Fig. 3.

To be able to make a correct diagnosis of pathological conditions it is absolutely necessary that the observer be thoroughly acquainted with normal ones as well as such variations from normality as may be still classed as physiological.—*Zeitshift für Vergleichende Augenheilkunde. Vol. I., No.* 2.

## Figures and Tables

**Fig I. f1:**
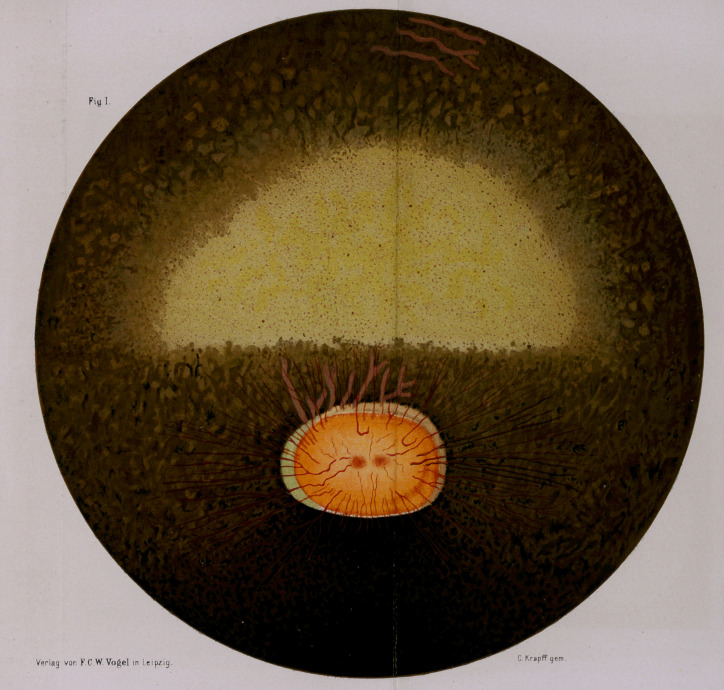


**Fig II. f2:**
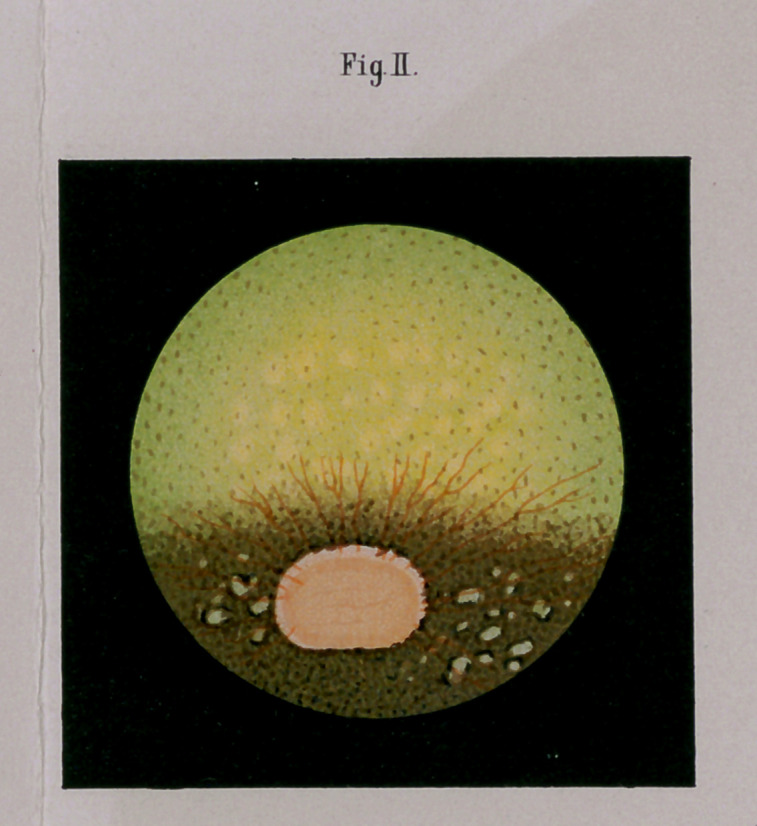


**Fig III. f3:**